# Atypical Polypoid Adenomyoma of the Vagina: Follow Up and Subsequent Evolution: A Case Report and Update

**DOI:** 10.3390/diagnostics12020368

**Published:** 2022-02-01

**Authors:** Melinda Ildiko Mitranovici, Ioan Emilian Oală, Izabella Petre, Marius Lucian Craina, Silviana Narcisa Floruț, Diana Maria Chiorean, Iuliu Gabriel Cocuz, Sabin Gligore Turdean, Ovidiu Simion Cotoi, Lucian Pușcașiu

**Affiliations:** 1 Department of Obstetrics and Gynecology, Emergency County Hospital Hunedoara, 14 Victoriei Street, 331057 Hunedoara, Romania; oalaioanemilian@gmail.com; 2 Department of Obstetrics and Gynecology, Victor Babeș University of Medicine and Pharmacy, 2 Eftimie Murgu Sq, 300041 Timisoara, Romania; petre.izabella@umft.ro (I.P.); mariuscraina@hotmail.com (M.L.C.); 3 Department of Pathology, Emergency County Hospital Hunedoara, 14 Victoriei Street, 331057 Hunedoara, Romania; florutsilviana@yahoo.com; 4 Department of Pathophysiology, George Emil Palade University of Medicine, Pharmacy, Science, and Technology of Târgu Mureș, 38 Gheorghe Marinescu Street, 540142 Târgu Mureș, Romania; chioreandianamaria@yahoo.com (D.M.C.); iuliu.cocuz@umfst.ro (I.G.C.); sabiturdean@yahoo.com (S.G.T.); ovidiucotoi@yahoo.com (O.S.C.); 5 Department of Pathology, County Clinical Hospital of Târgu Mureș, 540072 Târgu Mureș, Romania; 6 Department of Obstetrics, Gynecology County Emergency Hospital, University of Medicine and Pharmacty Targu Mures, 38 Gh. Marinescu Str., 540142 Targu Mures, Romania; puscasiu@gmail.com

**Keywords:** atypical polypoid adenomyoma, atypical endometrial hyperplasia, carcinosarcoma, total hysterectomy with bilateral salpingo-oophorectomy, tumor resection

## Abstract

Atypical polypoid adenomyoma (APA) is a rare tumor developed from a mix of cells of epithelial and mesenchymal origin. We present the case of an 84-year-old patient with atypical polypoid adenomyoma on the vaginal vault, after total hysterectomy with total adnexectomy for endometrial hyperplasia with atypia four years ago. Not following regular indicated gynecological appointments, the symptoms presented were vaginal bleeding and anemia. The importance of the case consists both in the unique way in which the adenomyoma appears on the vaginal vault and in the subsequent evolution of this pathology. After complete resection, it recurs in five months with a malignant transformation into carcinosarcoma. This fact shows that adenomas can turn not only into carcinomas but also the mesenchymal component can progress to sarcoma, a fact of exceptional rarity. Follow-up and accurate diagnosis are essential for proper management, which is a challenge anyway due to the lack of case studies.

## 1. Introduction

Atypical polypoid adenomyoma (APA) is an uncommon polypoid lesion located in the lower uterine segment or endometrial cavity, often diagnosed on biopsy or curettage. Reported first by Mazur in 1981, it has the potential of malignant transformation in adenocarcinoma and a high incidence of recurrence, but does not metastasize [1,2,3,4]. It is a rare tumor, and its diagnosis is usually missed. The age range for its occurrence is 21–73 years. So far, 237 cases have been reported worldwide. Being a rare condition, the experience with its diagnosis and optimal management is limited. Its management should be individualized according to age, clinical features, and fertility desires [5].

## 2. Case Presentation

We present the case of a post-menopausal 84-year-old G5P1 morbidly obese woman with a body mass index (BMI) higher than 35 who presented in March 2021 to the Obstetrics and Gynecology department of our clinic, with the main complaint of vaginal bleeding. Medical history included high blood pressure and asthma and the patient was not under hormone replacement therapy (HRT). In her past medical history, the patient had a total hysterectomy with bilateral salpingo-oophorectomy by laparotomy in 2017 for severe vaginal bleeding. The pathological report of the hysterectomy specimen showed complex hyperplasia of the endometrium with atypia, nests of typical adenomyosis consisting of intramural aggregates of benign endometrial stromal cells with scattered endometrial glands, an intramural leiomyoma, a fibromatous cervix, and large ovarian calcifications secondary to a calcified corpus albicans, such as epithelial inclusion cysts. Subsequently, she developed a surgical site breakdown of the skin and subcutaneous tissues and was secondary sutured. For personal reasons, the patient had decided not to pursue further investigations.

During her visit four years later, the medical examination showed a normal abdominal status. Nevertheless, the speculum and vaginal examination revealed a solid mass of the vaginal vault. A transvaginal ultrasound showed a solid heterogeneous tumoral mass, which measures over 100 mm diameter.

There was no free fluid in the peritoneal cavity. Under neuroleptic anesthesia and paracervical block, we resected with difficulty the pediculate tumor to its 10 mm implantation base by electrocauterization procedure, with 5 mm margin, in oncological safety limit, using a loop resection and the resulted sample was sent further to the histopathology department, for histological evaluation.

On laboratory testing, a complete blood cell count (FBC) was notable for: Hemoglobin—10.5 g/dL, hematocrit—35.2%, platelets—400 × 109/L, the number of leukocytes (WBC) as 13.14 × 109/L, limphocytes—6.9%, neutrophiles—87.3%. Biochemistry showed that the hsCRP level was 91.20 mg/L, with an erythrocyte sedimentation rate (ESR)—60 mm/h. The vaginal swab culture revealed vaginal colonization with *E. coli*-Cefoperazone/Sulbactam sensitive.

**Figure 1:**
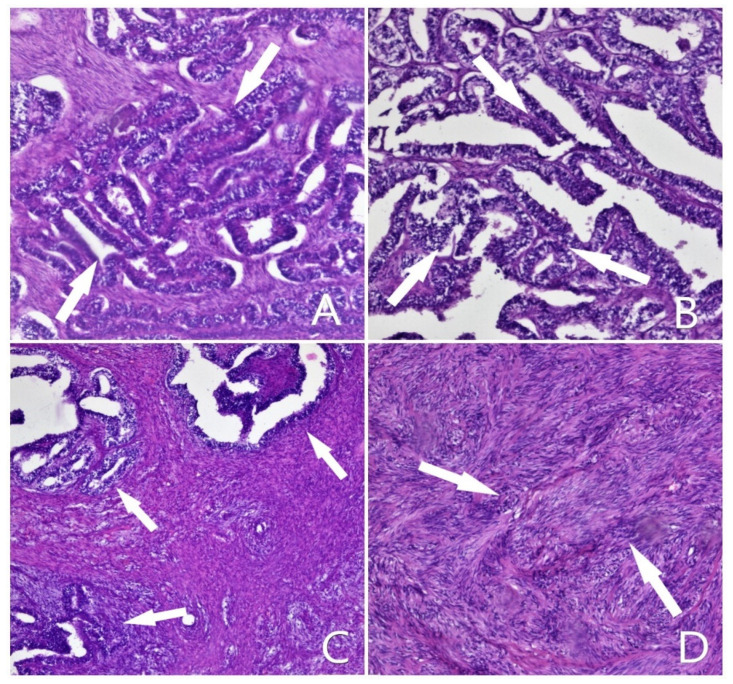
(**A**,**C**) Hiperplastic endometrial glands, arranged ”back-to-back”, in a dense, myomatous stroma–HE (Hematoxiline and Eosine), ob. 10×; (**B**) Epithelial component–in detail–HE, ob. 20×; (**D**) Mezenchimal component, without atypia–detail–Hematoxiline and Eosine (HE), ob. 10×. (Indicated by arrow).

Histopathology of the specimen revealed atypical polypoid adenomyoma (Figure 1). The histologic criteria used for this diagnosis were: The presence of architecturally and cytologically atypical endometrial glands, separated by smooth muscle cells’ intersecting fascicles, increased cellularity and different architecture from that of the normal myometrium of the stromal component, the lesion had a well-demarcated, ”pushing” margin. There were no immediate complications, and the patient was safely discharged.

Multidisciplinary discussions between oncologists and other gynecologists have been followed in order to establish a correct treatment (GnRh analogues or radiotherapy). Due to the lack of an effective treatment protocol, we decided to follow up by further evaluation.

**Figure 2:**
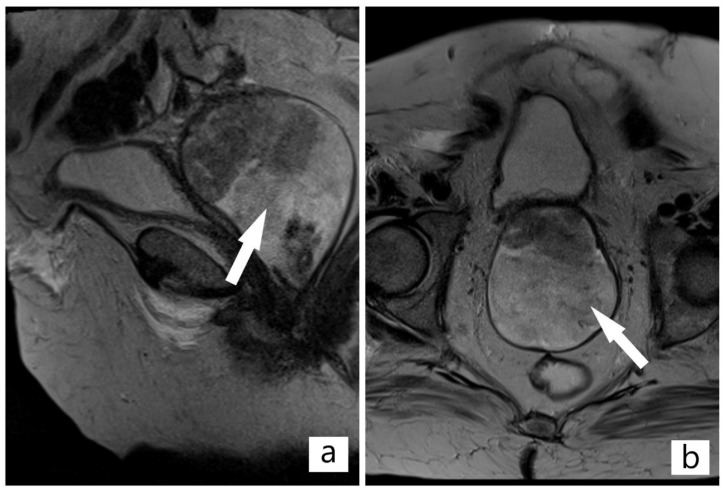
(**a**,**b**) Magnetic resonance imaging (MRI) of the pelvis: Solid mass located at the vaginal vault, intensly gadophilic, extending to the lower 1/3 part of the vagina, no direct infiltration of the urinary bladder (Arrow indicates the tumor).

After five months, the patient returned to our department and reported increased vaginal bleeding. On laboratory testing, the complete blood cell count (FBC) revealed a notable panel for: Hemoglobin—9.7 g/dL, hematocrit—33.7%, leukocytes (WBC)—16.15 × 10^9^/L, trombocytes—484 × 10^9^/L. The biochemistry part has undergone several changes comparative to the previous admission: Uric acid—7.81 mg/dL, blood glucose level—117 mg/dL, creatinine—1.71 mg/dL, hsCRP level—206.53 mg/L. The vaginal swab culture revealed vaginal colonization with *Proteus mirabilis*-gentamicin and cephalosporins sensitive. The transvaginal ultrasound examination showed a solid mass with similar characteristics as the previous one identified in March. Magnetic resonance imaging (MRI) of the pelvis revealed a 100 × 71 × 80 mm (craniocaudal × anteroposterior × transverse) mass located at the vaginal vault, intensely gadophilic, extending to the lower 1/3 part of the vagina (Figure 2a).

A part of the tumoral mass closely abutted the urinary bladder, with no evidence of direct infiltration (Figure 2b). On craniocaudal examination, the tumor erases the demarcation line of the sigmoid colon without penetrating it. No pelvic and inguinal level lymphadenopathies were observed. The urinary bladder did not show obvious signs of parietal lesions. The rectum revealed a normal appearance. After multidisciplinary discussions, transvaginal surgical resection of the tumor to its 50 mm wide implantation base, extended to the anterior vaginal wall, with 5 mm margin, using a loop electrosurgical excision, was decided and immediately performed. The evolution was favorable and the patient was discharged.

**Figure 3:**
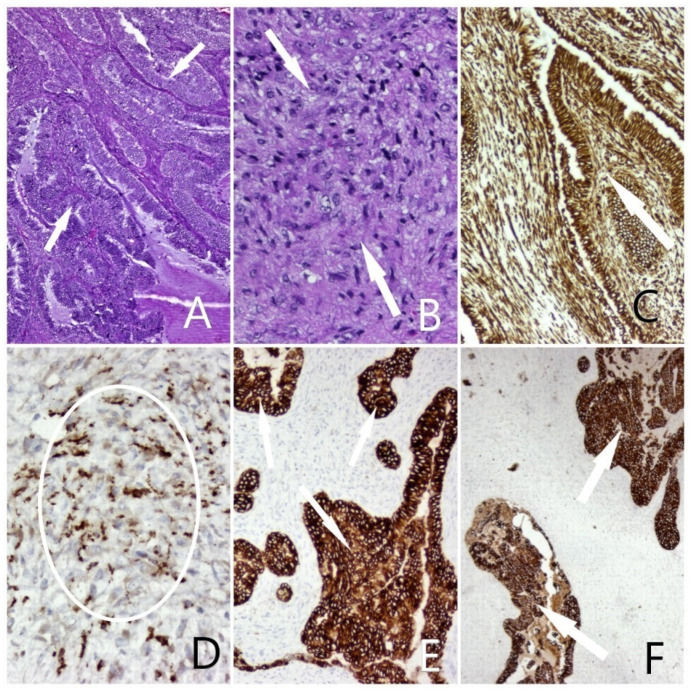
(**A**) Carcinosarcoma, epithelial component: Endometrial adenocarcinoma, atypical glandular structures, lined by a columnar epithelium–HE, ob. 5×; (**B**) Carcinosarcoma, mesenchymal component: Storiform proliferation of mesenchymal cells with marked anisocytosis, anisocaryosis, pleomorphism, hyperchromic nuclei–HE, 20×; (**C**) Biphasic tumoral positivity–Vimentin positive staining, ob. 5×; (**D**) Malignant mesenchymal cellular elements–CD68 positive staining, ob. 20×; (**E**) Epithelial malignant component-CTK AE1/AE3 positive staining, ob. 10×; (**F**) Epithelial malignant component–CK7 positive staining, ob. 10× (Lesions indicated by arrows).

Subsequently, the surgical specimen was sent further to the pathology department for histological and immunohistochemical evaluation. Routine histological stain (Hematoxiline and Eosine) revealed a biphasic tumor proliferation, corresponding to a moderately differentiated endometrioid adenocarcinoma (40%), along with a tumor proliferation with the appearance of high-grade sarcoma (60%). The epithelial component was composed of solid areas and agglomerated glandular and villoglandular atypical structures, with a reduced stroma and present desmoplastic reaction. The glandular structures were delimited by a pseudostratified and multilayered cylindrical epithelium, with marked cyto-nuclear atypia (modified nucleus/cytoplasm ratio in favor of the nucleus, hyperchromatic nuclei, and frequent typical and atypical mitotic figures). The mesenchimal component was composed of a tumoral proliferation with storiform character, with round or elongated cells, spindle-shaped, with nuclei with marked pleomorphism, with coarse chromatin and prominent nucleoli. Areas of necrosis were present, and no images of lympho-vascular invasion were identified (Figure 3).

The results of immunohistochemical techniques, using antibodies against CD68, Vimentin, CK AE1/AE3, and CK7 were consistent and confirmed the diagnosis of high-malignant carcinosarcoma.

The patient was recalled for the management of carcinosarcoma and presented, during admission, a basicervical femoral fracture of the right hip, due to osteoporotic bone. We requested a computer tomography (CT) scan, which revealed the suspicion of bone metastasis and tumor recurrence. An oncological commission discussed the case, and the patient was sent to radiotherapy, although there was no evidence of a treatment protocol for this type of cancer.

## 3. Discussion

Based on publication data, atypical polypoid adenomyoma (APA) is an endometrial tumor with low malignant potential and low incidence rate, which generally occurs in patients of reproductive age. Being considered as an indicator or precursor for endometrial endometrioid adenocarcinoma or coexisted with endometrioid adenocarcinoma [6,7,8], it should be treated from the beginning as a potential-aggressive tumor in order to avoid delays in therapy. Moreover, it is mandatory to inform the patient about all these risks. Matsumoto et al. reported that the majority of the patients diagnosed with APA were nulligravida (75.9%) and nullipara (86.2%) [5,9,10]. Many studies reported a possible relationship between hormones and APA. Its pathogenesis has been reported to be related with estrogen-related factors, obesity, diabetes mellitus, prolonged estrogenic stimulation, and hormone replacement therapy (HRT). It was not the case. As an update, we have to take in consideration other patients with vaginal bleeding who could be an exception. The precise mechanism has yet to be understood [9,11,12,13]. In spite of the clinical, pathological, and therapeutical features of this tumor, our case represents a rarity, due not only to its exceptional occurrence at the level of the vagina, but for its malignant transformation of both, epithelial and mesenchymal components. Moreover, even the complete removal of the tumor has been demonstrated as being inefficient as therapy.

I chose to present here a classification of adenomyoma according to the World Health Organization (WHO) 2014, which includes: Atypical polypoid adenomyoma (APA), endometrial-type adenomyoma (EA), and endocervical-type adenomyoma [7,14,15]. We can conclude that it does not include the atypical polypoid adenomyoma of vaginal vault-type, developed after complex hyperplasia of the endometrium with atypia. As an update, it is important that WHO’s classification of adenomyoma is to be extended. To our knowledge, there is no case described in the literature, and its occurrence is likely due to contamination of the vaginal vault with atypical cells during the hysterectomy procedure.

C. Huang et al. suggest that endometrial evaluation should be carefully performed in patients with post-menopausal bleeding, it being considered a negative prognostic factor for subsequent uterine disease, as in our patient’s case. Moreover, he admits hysteroscopy as being a very successful procedure in the detection of endometrial lesions [7,16]. Evaluation of the endometrium by hysteroscopy D and C raises the accuracy in diagnosis of endometrial cancer [7,17,18,19,20,21,22].

Denschlag and Urlich declared that there are no typical laboratory testing abnormalities associated with the diagnosis of carcinosarcoma, although anemia is present in up to 10% as a result of vaginal bleeding, also an important aspect of our case presentation [23,24,25]. On the other hand, Huang et al. suggest that preoperative elevation of the epithelial tumor marker CA-125 may highlight an advanced stage of the disease [24,26].

**Figure 4:**
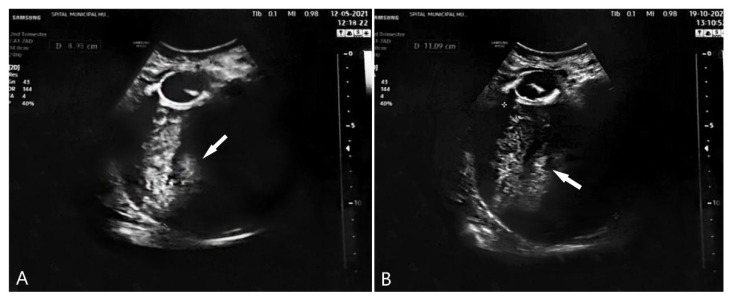
(**A**) Ultrasound examination of atypical polypoid adenomioma (APA); (**B**) ultrasound examination of carcinosarcoma.(Indicated by arrow).

Of particular importance is a proper pathological-anatomical work-up, uterine carcinosarcoma being ultimately a histological diagnosis. It is not possible to differentiate a carcinosarcoma from an endometrial carcinoma or uterine sarcoma based on symptoms, imaging, or laboratory findings only [24,25]. This is why in our case, histopathological specimen was re-tested in another clinic. As in our patient’s case, the ultrasound image of atypical polypoid adenomyoma and carcinosarcoma are very similar (Figure 4).

Histopathologically, the mechanism of malignancy is not well-known [20,21,22,23]. Matsumoto et al. found adenocarcinoma type malignancy after atypical adenomyoma treatment. Recurrence occurs in 1–10% of cases [7,21,22]. Therefore, follow-up is extremely important. McCluggage describes a very rare situation of epithelial neoplasm of the uterus that underwent sarcomatous transformation, the epithelial component being the driving force, it can be considered a subtype of uterine epithelial malignancies and not a subtype of uterine sarcoma [14]. However, to our knowledge, there is no case described in the literature of transformation into sarcoma of an atypical polypoid adenomyoma of the vaginal vault.

In terms of treatment, surgery represents the first line of therapy of uterine carcinosarcoma, staged according to the 2017 International Federation of Gynecology and Obstetrics (FIGO)/Tumor, Node, Metastasis (TNM) classification system. Complete surgical staging includes: Total hysterectomy, bilateral salpingo-oophorectomy, pelvic and para-aortic lymph node dissection, and cytology of the peritoneal washing [24,27,28]. Pelvic and para-aortic lymphadenectomy may also be associated with improved overall survival [24,29,30]. However, it is not the case. Multidisciplinary discussions suggest that transvaginal surgical resection would be the main management, no lymphadenopathies were observed on MRI. Lack of data in the literature regarding vaginal carcinosarcoma prevents us from establishing coherent management of this pathology. In order to treat with GnRh analog, only a single case that responded favorably was reported in the literature [7,8].

## 4. Conclusions

We intend to highlight the importance of careful medical approach regarding the evolution of atypical polipoid adenomyomas and we have to emphasize the importance of an adequate histological evaluation and coherent management of this pathology. In our case, this type of tumor showed rapid malignant transformation, even if our patient did not use hormone replacement therapy. Even more so, our case represents a rarity, due to its exceptional occurrence at the level of the vagina. Moreover, the mesenchymal component malignancy of this particular type of atypical polypoid adenomyoma into carcinosarcoma is not mentioned in the literature, to the best of our knowledge.
